# Inert-Atmosphere Microfabrication Technology for 2D Materials and Heterostructures

**DOI:** 10.3390/mi15010094

**Published:** 2023-12-31

**Authors:** Aliaksandr Duleba, Mikhail Pugachev, Mark Blumenau, Sergey Martanov, Mark Naumov, Aleksey Shupletsov, Aleksandr Kuntsevich

**Affiliations:** 1 P.N. Lebedev Physical Institute of the Russian Academy of Sciences, Moscow 119991, Russia; a.dulebo@lebedev.ru (A.D.); m.pugachev@lebedev.ru (M.P.); m.blumenau@lebedev.ru (M.B.); martanovsg@lebedev.ru (S.M.); shuplecovav@lebedev.ru (A.S.); 2 Dukhov Research Institute of Automatics (VNIIA), Moscow 127055, Russia; mark.naumov1999@gmail.com

**Keywords:** van der Waals heterostructures, 2D materials, lithography, nanofabrication, metal films

## Abstract

Most 2D materials are unstable under ambient conditions. Assembly of van der Waals heterostructures in the inert atmosphere of the glove box with ex situ lithography partially solves the problem of device fabrication out of unstable materials. In our paper, we demonstrate an approach to the next-generation inert-atmosphere (nitrogen, <20 ppm oxygen content) fabrication setup, including optical contact mask lithography with a 2 μ m resolution, metal evaporation, lift-off and placement of the sample to the cryostat for electric measurements in the same inert atmosphere environment. We consider basic construction principles, budget considerations, and showcase the fabrication and subsequent degradation of black-phosphorous-based structures within weeks. The proposed solutions are surprisingly compact and inexpensive, making them feasible for implementation in numerous 2D materials laboratories.

## 1. Introduction

Van der Waals heterostructures offer a new paradigm for electronic device development. By stacking individual layers of 2D crystals with precise control over interlayer twist angles, thicknesses, and compositions, researchers can create metamaterials with tailored properties [1,2]. This approach enables the creation of novel electronic devices with unprecedented functionality [3,4,5,6].

Unfortunately, most 2D materials, except for graphene and its compounds, hexagonal boron nitride, and some transition metal dichalcogenides, like MoS_2_, have a tendency to degrade in air with time [7]. Assembling van der Waals heterostructures in the inert atmosphere of glove boxes [8,9,10,11], and even in high vacuum [12,13], is crucial for working with unstable-in-air 2D materials. To prototype a device in an inert atmosphere, the stack is assembled and positioned on the substrate inside the glove box. A properly fabricated heterostructure can then be used under ambient conditions. Microscope- and micromanipulator-based devices are used for this purpose [14]. As a rule, the structure should have an air- and chemistry-stable top layer, e.g., graphene or hexagonal boron nitride. Further processing, such as mesa-etching, fabrication of the gate electrode, or contacts to the active layer, is carried out ex situ, using techniques including lithography, plasma or wet etching, coatings, and others. This route is typical for numerous 2D materials laboratories [15].

Implementing ex situ operations within an inert atmosphere would significantly enhance researchers’ capabilities. For example, more intricate mesa-formations would be possible, or the capping layer could be excluded.

There have been attempts to build such a complex of equipment, e.g., “clean room in the glove box” in Ref. [16]. This solution includes several connected glove boxes with various technological processes. However, such a setup is complex and prohibitively expensive for a standard laboratory. While assembling stacks in glove boxes is a standard practice, lithography and other operations present challenges. Scanning probe techniques could serve as a powerful inert atmosphere tool for 2D materials. State-of-the-art manipulation with the flakes and heterostructures, including scratching lithography and welding, is demonstrated in Refs. [17,18]. Moreover, glove-box-compatible scanning probe lithographers are already commercially supplied by Heidelberg Instruments (Accessed on 30 December 2023). Such a solution is, of course, quite expensive and has low throughput compared to optical lithography.

Additionally, there are ongoing efforts to manufacture micro-devices from 2D flakes without the need for lithography within the glove box environment. For instance, reference [19] proposes the use of pre-patterning via contacts in hexagonal boron nitride (hBN) outside the inert atmosphere. Similarly, reference [20] suggests defining the shape of the flakes using a pre-patterned adhesive matrix, indicating that all lithographical operations are carried out outside the inert atmosphere. These alternative methods demonstrate the potential to advance fabrication processes for 2D materials while circumventing the need for lithography within inert environments.

We were encouraged to simplify the “clean room in the glove box” concept and propose a more cost-effective solution. Our key idea involves utilizing the same compact home-made setup for both stack assembly and mini-contact mask lithography [21]. For metallization processes, sputtering, or plasma etching we use a reconfigurable vacuum chamber with direct access from the glove box. For liquid processes we use a second glove box connected to the main one through the load lock. Our system also enables the transfer of samples for resistivity measurements in the inert atmosphere: for this purpose, the glove box is equipped with a microscope to mount the sample onto the measurement socket.

In this paper, we first consider the general scheme of the setup, followed by a detailed examination of each component and its key features. We conclude by demonstrating the proof of functionality. We aim to inspire other researchers to construct their own home-made all-inert-atmosphere technological units, hence should facilitate experimental studies of air-unstable 2D materials, such as MXenes [22], chalides [23] and pnictides [24], perovskites [25], chalcogenides, and so on.

## 2. Overview of the Technology

**Figure 1:**
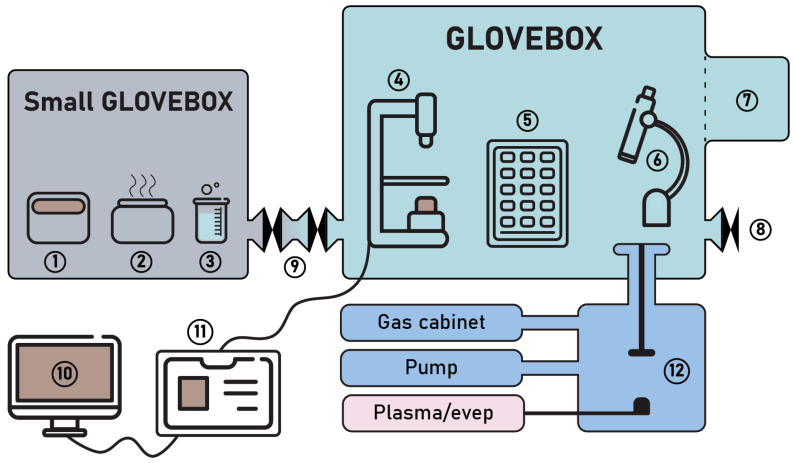
Schematic of the setup. 1. Spin-coater; 2. hot plate; 3. liquid processes; 4. VdW assembly + lithography; 5. storage; 6. long-focus microscope; 7. load lock; 8. transfer to cryostat; 9. small load lock; 10. computer; 11. control block; 12. vacuum chamber.

**Figure 2:**
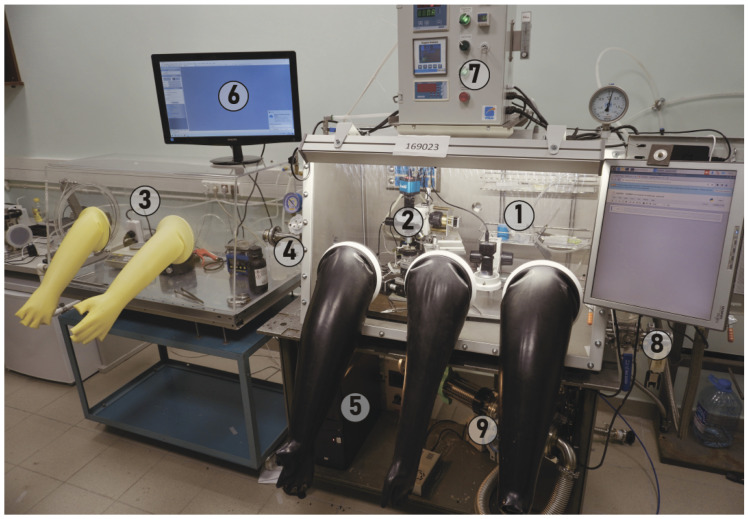
Photo of the setup. 1. Glovebox; 2. VdW assembly + lithography; 3. small glovebox; 4. small load lock; 5. computer; 6. screen; 7. control unit outside; 8. transfer to cryostat; 9. vacuum chamber.

The general scheme of the setup and its photo are shown in Figure 1 and Figure 2, respectively. Functional blocks are shown in different colors and described in different subsections below.

Two gloveboxes are necessary to split “clean” processes, where much more controllable conditions are required, and “dirty” processes in the small glovebox, with overheating and chemistry.

### 2.1. Glovebox

**Figure 3:**
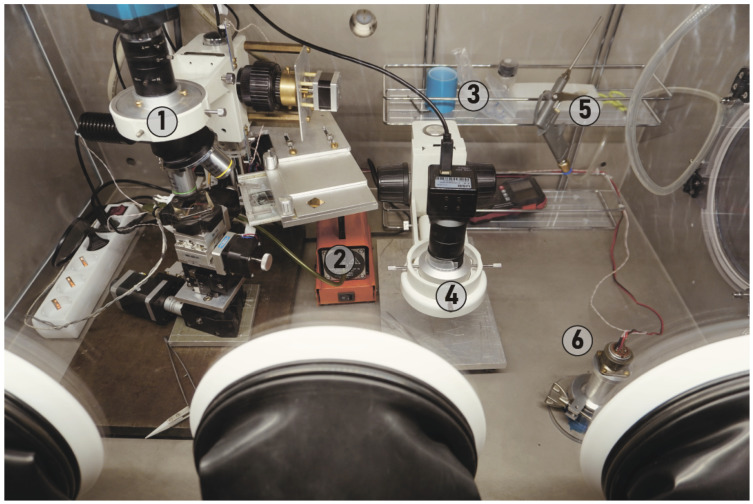
Photo of the glovebox inside. 1. The transfer lithography machine; 2. pump for samples fixation; 3. storage; 4. long-focus microscope; 5. nitrogen by pressure; 6. glovebox and vacuum chamber connection flange.

We use a 1.1 × 0.7 × 0.8 m^3^ nitrogen glovebox with a gas purification system and three gloves. The glovebox is equipped with a load lock (# 7 in Figure 1). A home-made transfer machine combined with contact mask optical lithography is the key component of the glovebox (marked by # 4 in Figure 1; see detailed discussion in Section 2.3). A second microscope with low magnification and long focal length (marked by # 6 in Figure 1) is necessary for visual inspections, with manual gluing of the contacts to the pads (see example in Figure 7f). Inside the glovebox there is also storage for the components (crystals, substrates, samples, tapes) and tools, including scissors and tweezers (# 5 in Figure 1). We do not place the computer inside the glovebox to reduce heat dissipation and problems with service. Instead, we feed numerous cables through the wall of the glovebox. Special care should be taken to avoid gas leakage through the wires. For this purpose, we used a sealing kit by Roxtec (Accessed on 30 December 2023). A special KF40 port allows samples to be transferred from the glovebox directly to the measurement device (# 8 in Figure 1). The components of the glove box are shown in Figure 3.

### 2.2. Wet Process (Small) Glovebox

Working with photoresists requires a hot plate and a spin-coater that should be generally avoided in the glovebox. Moreover, chemicals are stored here. We do not use a gas purification system in the small glovebox, because the sample is not exposed here for a long time. For the same reason, we do not cover this glovebox with yellow or orange UV protection. At the same time, photoresists are stored in brown boxes to avoid UV exposure. The major considerations here are:

- compact size of the hot plate to avoid overheating;
- compact size of the spin-coater;
- AC mains inside;
- home-made KF40 load lock to pass the samples to/from the main glovebox. The samples are transferred in Petri dishes;
- USB cable is fed through the wall to connect the USB temperature/moisture monitor;
- chemicals (acetone, isopropanol, photoresist, water, wet etching) are stored in the closed bottles;
- the glovebox is equipped with tweezers, pipettes, etc.

In our particular case, we built a nitrogen pressure stabilization system ourselves out of pressure controller ZSE30AF-01-A-L (Accessed on 30 December 2023), 24 V DC/ 220 V AC relays from Aliexpress (Accessed on 30 December 2023). These are used to control 220 V electromagnetic valves Aliexpress(Accessed on 30 December 2023). A 24 volt DC supply is taken from an AC/DC converter from Aliexpress (Accessed on 30 December 2023). The price of all components for pressure stabilization is thus below USD 100.

### 2.3. Transfer Lithography Machine

One of the central ideas of the present paper is to combine two crucial components in a simple home-made metallographic microscope:

- Transfer machine to assemble heterostructures out of separate layers;
- Small mask aligner to make lithographical operations.

Historically, the first transfer machines for van der Waals heterostructures were mask aligners [26]. However, they were too big to be placed in a glove box. Much more compact, home-made machines were then designed. In fact, such a machine is an XYZ manipulator, where the substrate is placed on the heated stage within the field of view of the metallographic microscope. An additional holder is needed for the temporary substrate, which is usually a glass slide with some organic droplets—a carrier of heterostructures. This glass slide is positioned between the substrate and the objective, and the focus switches between the substrate and the surface of the droplet during the transfer. In our case, both the microscope and substrate stages are motorized (refer to Section 2.5 for more details) to eliminate the need for manual adjustments with micrometers. Additionally, observing the microscope field of view through a remote monitor is hardly compatible with manual handling.

**Figure 4:**
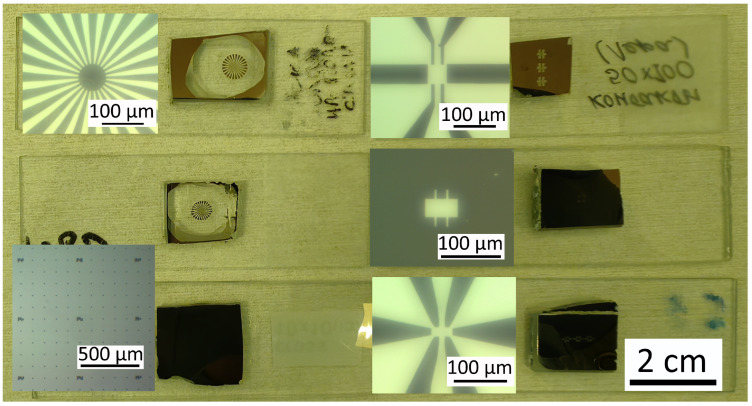
A set of masks for making mesa-structures, contacts, and labels.

The metallographic microscope is equipped with a 4-position objective turret. Three objectives are used (5× NA 0.15, 10× NA 0.25, and 20× NA 0.4). The fourth position contains an LED, which is used for exposure of the photoresist through the shadow contact mask in the regime of the lithographer. The resolution of such mask lithography is below 2 μ m and the precision of alignment is about 1 μ m. The masks are manufactured out of ready-made electrochemically chrome-coated glass plates. We employ direct laser writing photolithography and liquid chromium etching to define the design. A library of standard masks (shown in Figure 4) enables us to perform lithography on samples of flakes and heterostructures of various shapes and sizes. In more detail, the processes of mask fabrication and lithography are described in Ref. [21].

### 2.4. Vacuum Processes

**Figure 5:**
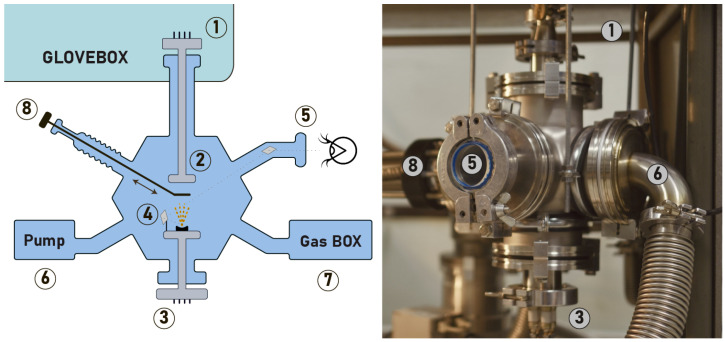
Schematic (left panel) and photo (right panel) of the vacuum chamber. 1. Hermetically sealed connector; 2. a platform for the samples; 3. four current-carrying electrodes; 4. evaporation boat; 5. watchport; 6. turbo pump; 7. gas supply station; 8. damper and electrode to ignite the plasma.

The vacuum system should be compact, cheap, and versatile. To simplify the customization we use a 6-fold ISO100 flange as a chamber. It is connected to the glove box with bellows and fixed with water-pipe clamps to the bottom wall of the table, where the glovebox is located, as shown in schematic view in Figure 5.

A sample rod (# 1) is fed through the bellows from the glovebox. There is a platform for the samples at one end of the rod (# 2). At the other end, there is a hermetically sealed connector. A heating ceramic element is attached to the platform, which can heat the platform up to 200 degrees Celsius. The temperature is controlled with a thermocouple inserted inside the platform and a proportional–integral–derivative (PID) regulator from the microcontroller.

Four current-carrying electrodes used for chromium/gold thermal evaporation are fed through to the bottom of the chamber (# 3). The corresponding feedthroughs are available, e.g., from Aliexpress (Accessed on 30 December 2023). The conductors are attached to a holder, which can be used for both a chromium rod and an evaporation boat (in our case with gold). The third ISO flange has a watchport to control the evaporation process (# 5) with a mirror (# 4).

Two more flanges of the chamber are used to supply various gases (O_2_, SF_6_, and Ar in our case) through the leak valves or MFC (# 7), and a Pfeiffer HiCube Eco 80 turbopump (# 6). The last (6th) flange is used both as a damper above the evaporation cell and as an electrode to ignite the plasma near the sample (# 8). A 500 W 13.56 MHz generator is used for the plasma generation.

Hence, the system facilitates cleaning the substrate in a plasma environment, transferring the sample from an inert atmosphere to a vacuum, annealing it if needed at temperatures up to 200 degrees Celsius, thermally evaporating two metals, and then returning it to the inert atmosphere. This cycle, coupled with the availability of liquid processes in a small glovebox, allows for a closed-loop lithography inside an inert atmosphere.

### 2.5. Automation

**Figure 6:**
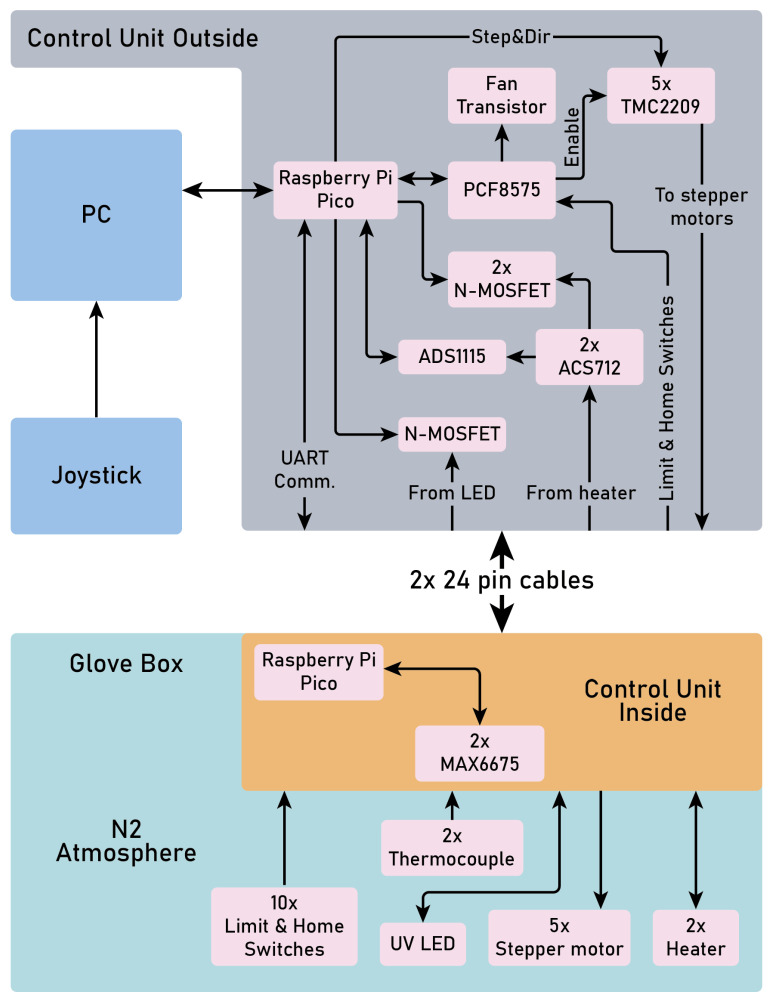
Schematic of the automation control setup.

A simple schematic of the automation control is presented in Figure 6. It consists of three main parts:

- A computer for users to control the setup with a joystick or keyboard/mouse;
- Outside control unit, located near the PC;
- Inside control unit, located in the glovebox.

The idea is to place the unnecessary heat-generating components, such as stepper motor controllers, outside the glovebox for better thermal management. Two control units are connected with two 24-pin cables.

We employ an XYZ Θ stage equipped with stepper motors. The microscope has a stepper motor for autofocus. To control these components, we utilize a Raspberry Pi Pico microcontroller along with TMC2209 controllers. The TMC2209 controllers operate in STEP/DIR control mode, utilizing En, Step, and Dir pins. While this mode offers limited functionality compared to the universal asynchronous receiver/transmitter (UART) mode, it ensures full compatibility with more affordable A4988 controllers.

For the XY stages, three Hall effect position sensors are implemented to manage endpoints and the home position. The Z stage and autofocus have upper and lower endpoints. Due to the various sensors and elements in use, the available pins on the Raspberry Pi Pico prove insufficient. To address this limitation, a PCF8575 I/O expander is employed. This expander takes charge of controlling endpoints and home position switches, managing the cooling fan through a transistor, and enabling or disabling the TMC2209 controllers via the En pin.

Apart from controlling the stepper motors, the microcontroller oversees various aspects of the transfer machine. To maintain the substrate’s temperature during the transfer process, a thermocouple with an MAX6675 chip is employed. This system is connected to a second Raspberry Pi Pico situated within the glove box. Placing the MAX6675 externally and routing the thermocouple signal through lengthy wires resulted in frequent errors and inaccurate readings. Communication between the two Raspberry Pi Picos is facilitated through UART, and the microcontroller inside the glove box allows for potential functionality expansion.

Regulating integral heat generation in the heater is achieved using an N-channel MOSFET and a pulse-width modulation (PWM) signal. With a proportional–integral–derivative (PID) controller, this system ensures the maintenance of the user-selected temperature. To set current limits for different heaters sharing the same source and detect potential short circuits, an ACS712 sensor is utilized for current measurement. However, the sensor’s output can surpass the 3.3 V limit of the Raspberry Pi Pico ADC. Consequently, an ADS1115 ADC is integrated and connected via the I2C bus through a logic level converter. For vacuum processes, a similar system is deployed, differing only in the heater.

For the mask lithography, an N-channel MOSFET transistor and a PWM signal are used. Adjusting the PWM fill factor enables modification of the integrated diode illumination, allowing for customizable duration and intensity. The code for the microcontrollers is written in Micropython. A control script is written in Python and various libraries. For the user’s convenience, it is possible to work with the application both with a joystick and by writing commands on the keyboard.

## 3. Cost Effectiveness

Each element in the inert-atmosphere technology setup may vary in price. For example, the price of the cheapest spin-coater by Instras (Accessed on 30 December 2023) might be as small as USD 700, whereas the top-level spin-coaters from Laurell Technologies (Accessed on 30 December 2023) are at least 10 times more expensive.

The aim of the paper is to show that this technology could be rather affordable. In order to save money, we tried, where possible, to buy used components (e.g., on eBay), or standardized cheap solutions (on aliexpress.com).

Table 1 summarizes our expenses and is given for reference. We also suggest money-saving solutions (the last two columns). The table column “recommended solution” does not have references to eBay and shows only vendors of brand new equipment. Our total expenses were about USD 30k, which seems to be more than an order of magnitude cheaper than “clean room in the glovebox” from Ref. [16].

Note that using refurbished components reduces costs. For example, a used RF plasma generator could cost about USD 1500 on eBay. Similarly, a pre-owned glovebox is significantly more affordable than a new one, etc. Instead of a motorized one, one can use a manual home-made transfer machine, that costs about USD 1000–1500 [11,27,28]. This is less convenient because the manual operations are hindered in the glove box, but it is still acceptable. The same is true for the vacuum part.

The majority of components in the presented setup are typical for a standard 2D materials laboratory. Running costs increase only due to glove boxes and include gases, gloves, oxygen sensors, and some regular service. We use nitrogen gas from liquid evaporation (an evaporator is required), which is less than 1000 L of liquid N_2_ per year (USD 300 maximum). Filling the system with argon is several times more expensive (about USD 1000 per year). Gloves should also be changed regularly (USD 400/year). Thus, the running costs are about USD 1000–2000/year, which is much cheaper than the price of components (crystals, substrates, photoresists, chemistry, and so on).

We do not calculate job costs and consumable components. These values are given for estimatation purposes, and mean that within a standard research grant it is possible to build home-made inert-atmosphere 2D materials technology or upgrade the existing technology to the inert-atmosphere state.

## 4. Examples of Operation

### 4.1. hBN-Packed Black Phosphorous Flake

**Figure 7:**
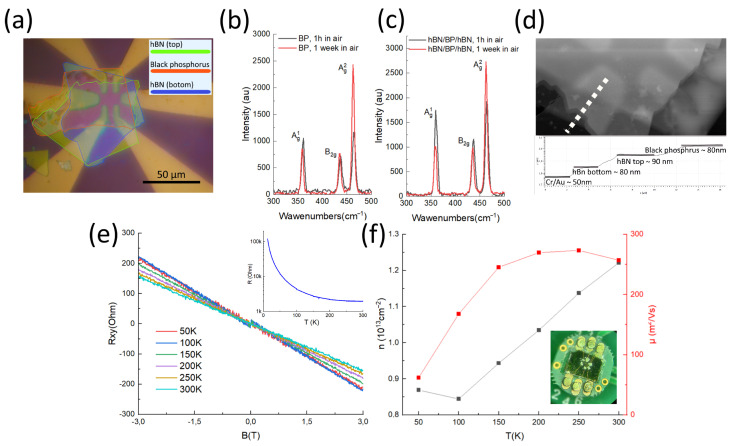
(**a**) Black phosphorus heterostructure hBN/BP/hBN obtained using the setup. Optical image of the heterostructure with metal contacts. (**b**,**c**) Raman spectra of BP after 1 h and 1 week in air, respectively. (**d**) AFM characterization of the hBN/BP/hBN heterostructure. hBN bottom ≈ 80 nm, BP ≈ 80 nm, and hBN top ≈ 90 nm. (**e**) $R_{xy}$ measurements of the heterostructure at different temperatures. The inset shows the resistance $R_{xx}$ as a function of temperature *T*. (**f**) Temperature dependence of the mobility and carrier density in a flake of black phosphorous. The inset shows a photo of the sample at the socket with silver paint glued contacts.

We fabricated an hBN/black phosphorus (BP)/hBN structure to demonstrate the performance of the glovebox. It has been shown that black phosphorus degrades in air [29,30,31] and an inert atmosphere is preferable. The hBN and BP flakes were mechanically exfoliated with Scotch Tape on a Si/SiO_2_ 285 nm substrate. Contacts were fabricated on top of a lower hBN flake using mask lithography and the thermal evaporation of chromium (≈10 nm) and gold (≈40 nm). Subsequently, BP/hBN was transferred on top of it using standard dry transfer methods. A photograph of the sample is shown in Figure 7a. The sample was removed from the glovebox for the AFM measurements (Figure 7b), Raman spectroscopy (Figure 7c,d), and transport measurements (Figure 7e). The thicknesses of the flakes obtained by AFM were hBN bottom ≈ 80 nm, BP ≈ 80 nm, and hBN top ≈ 90 nm.

Raman spectra of BP were obtained using an Inspectr R532 Raman microscope with an excitation wavelength of 532 nm, 1800 L/mm grating, and a 40X/NA O.6 objective. Measurements were performed after 1 h (Figure 7c), and 2 weeks (Figure 7d) of the sample being in contact with air. The Raman modes were 360.2/358.8 cm^−1^, 437.3/435.2 cm^−1^, and 464.4/462.8 cm^−1^ for the A^1^*_g_*, $B_{g}^{2}$ , and $A_{g}^{2}$ for the sample after 1 h and 1 week in air, respectively. The ratio of the integrated intensity of the Raman modes depends on the sample thickness and orientation; however, the $A_{g}^{1}$ / $A_{g}^{2}$ ratio of the Raman modes can be used to qualitatively evaluate the degree of oxidation of BP. After an hour in the air, the ratios of the integrated peak intensities were 0.78 and 0.86 for the portion of the sample covered and not covered with hBN. This value corresponds to data reported in the literature [30,32], indicating that the oxidation degree of black phosphorus is quite small and hBN prevents oxidation. The peak ratios were 0.34 (without hBN) and 0.4 (with hBN) after one week in the air.

Transport measurements were performed in a Cryogenic mini-CFMS cryostat at temperatures of 4–300 K and magnetic fields up to 3 T. Figure 7e shows $R_{xy}$ (antisymmetrized) as a function of B at different temperatures. The inset in Figure 7e shows the temperature dependence of the resistivity. The mobility at low temperature (50 K) was approximately 100 cm^2^/V·s, and at room temperature it was 250 cm^2^/V·s. The temperature dependencies of the carrier density and mobility are shown in Figure 7f. The room-temperature mobility values correspond to the literature data for bulk black phosphorus [29,33]. The mobility increases from 300 K down to 200 K and then starts to decrease. This behavior is similar to that of other semiconductors; see, e.g., [34]. The initial increase could be explained by a combination of phonon and charge impurity scattering [35]. The carrier density decreases as the temperature drops, which means suppression of the activated carriers and the population of low-mobility localized states. The electron system is close to the localization threshold. The Fermi level is, therefore, located at the band gap, which is confirmed by the Hall density of ∼10^13^ cm^−2^, which corresponds to less than ∼10^11^ cm^−2^ electrons per layer, a very small value for black phosphorous [36].

We thus demonstrate the capability to assemble the structures of air-unstable materials in a glove box in combination with other processes such as lithography, contact evaporation, and mounting to the socket for measurements.

### 4.2. Black Phosphorous Degradation

Black phosphorous monolayer covered by hBN is believed to be a “gold standard” for the demonstration of inert-atmosphere transfer techniques. Our experience is that hBN has some finite permittivity for oxygen, and the structures degrade over time anyway.

**Figure 8:**
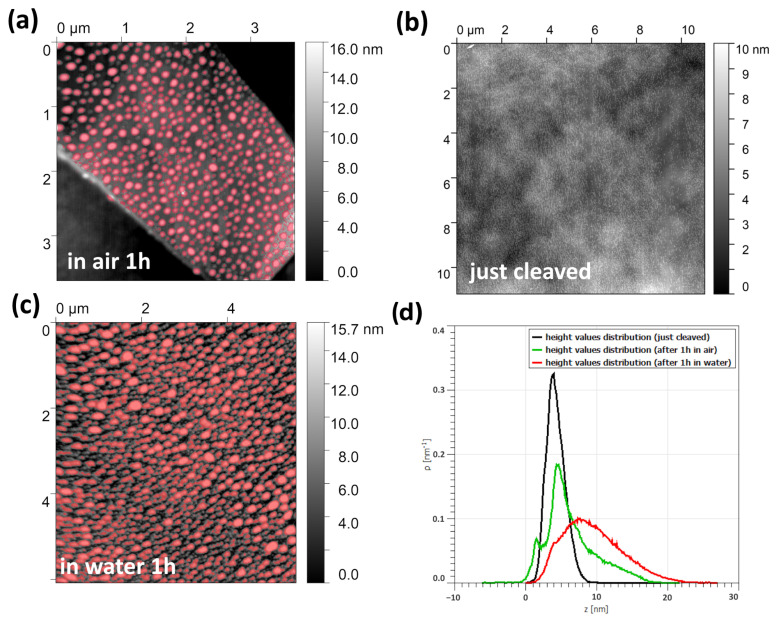
(**a**) AFM topography of black phosphorus after one hour under ambient conditions (bubbles are colored red). (**b**) AFM topography of the just-cleaved black phosphorus. (**c**) AFM topography of black phosphorus after one hour in deionized water (bubbles are colored red). (**d**) Comparison of height value distributions on the surface of BP: red, after 1 h in water; green, after 1 h in air; and black, just cleaved.

In this subsection, we exfoliate BP, fabricate some structures, and study the degradation factors. AFM ex situ images (acquisition time of 5 min) of the glove-box-stored and exfoliated black phosphorus flakes are shown in Figure 8a–c. We used the tapping mode of the Solver-47 scanning probe microscope by NT-MDT. The just-cleaved surface of BP has a certain roughness. After one hour of exposure in the air, a set of small bubbles appear on the surface. A similar flake stored for 1 h in the deionized water has a much more corrugated surface. The distribution of the deviations, shown in Figure 8d clearly show the dramatic difference between these three cases. We infer, therefore, that water is the key factor in the degradation.

**Figure 9:**
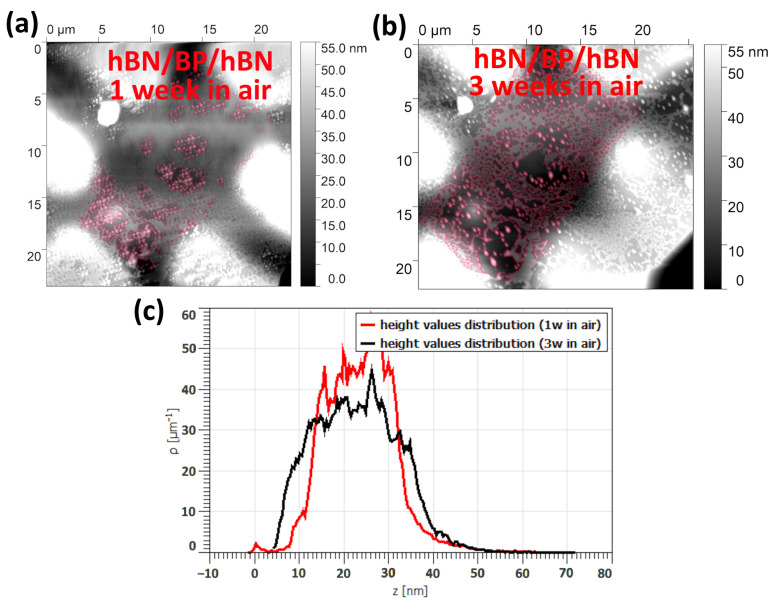
(**a**) AFM topography of the heterostructure hBN/BP/hBN with metal contacts after 1 week in air, with changes highlighted in purple. (**b**) AFM topography of the heterostructure hBN/BP/hBN with metal contacts after 3 weeks in air, with changes highlighted in purple. (**c**) Comparison of height value distributions on the surface of heterostructure hBN/BP/hBN: red, after 1 week in the air; black, after 3 weeks in the air.

To demonstrate that the degradation occurs even under the hBN layer, we stored an hBN-covered BP structure with bottom contacts under ambient conditions and performed AFM scans of the top hBN surface. The red color in Figure 9a,b shows the height changes observed after one and three weeks of exposure to air, respectively. The height distribution in these two cases is shown in Figure 9c. It is important to note that certain local areas did not exhibit degradation, indicating a probable tight adjustment between BP and hBN in these regions.

**Figure 10:**
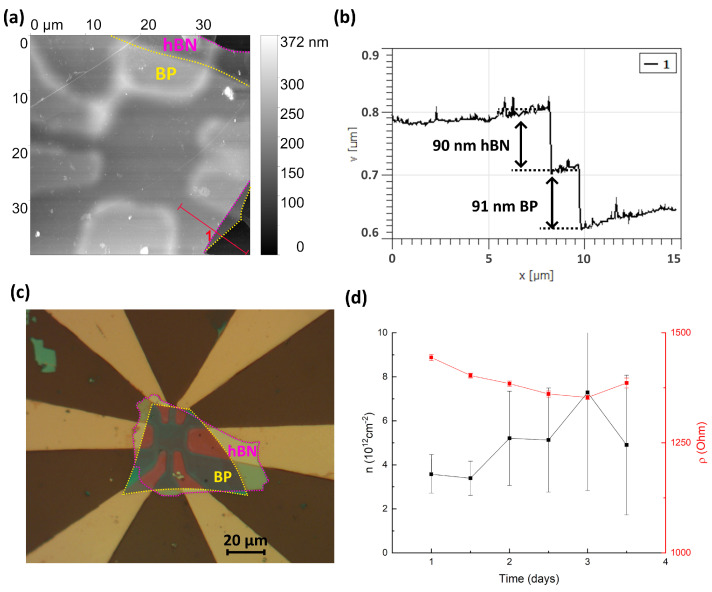
(**a**) AFM topography of the heterostructure hBN/BP with metal contacts. (**b**) Slice of line 1. The height of the BP is 91 nm, and the height of the hBN is 90 nm. (**c**) The optical image of the hBN/BP heterostructure. (**d**) Temporal variation in carrier density and resistivity for encapsulated black phosphorous structure (shown in the inset).

At the same time, for a rather thick flake (90 nm, shown in Figure 10) the transport properties do not exhibit any visible changes over time (see Figure 7). This means that in black phosphorous the degradation occurs mainly in the top layer and does not propagate deeply.

### 4.3. Contacts to Bismuth Chalcogenides

**Figure 11:**
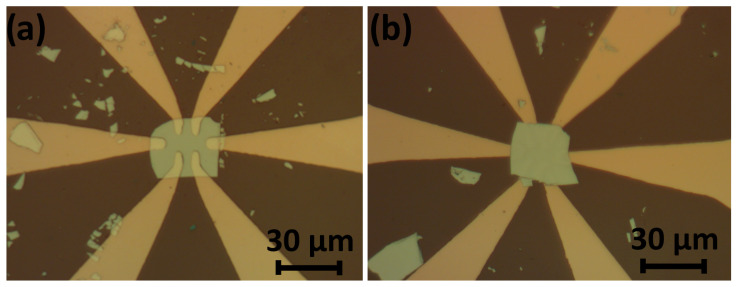
(**a**) Optical image of the Bi_2_Te_3_ flake under the metal contacts. (**b**) Optical image of the Bi_2_Te_3_ flake on the metal contacts.

To demonstrate the capabilities of the system, we fabricated contacts to the topological insulator Bi_2_Te_3_ (see Figure 11), which is one of the most studied topological insulators. The crystals are available from HQGraphene (Accessed on 30 December 2023). The surface of this material tends to degrade under air. If the crystal is stored for a considerable time under ambient conditions, the contacts will be highly resistive.

We fabricated contacts to Bi_2_Te_3_ flakes in two ways: (i) the flake of the crystal was first exfoliated to a substrate (oxidized Si wafer), and then top contacts (10 nm chromium/80 nm gold) were fabricated in our setup using contact mask lithography and lift-off, as shown in Figure 11a; (ii) the chromium/gold contacts were first fabricated on the substrate, and then, the flake was transferred atop, as shown in Figure 11b. The second route is similar to what we have done to black phosphorous.

Several (about three for each type) samples were fabricated. The typical thickness of a Bi_2_Te_3_ layer is about 100 nm. Two major characteristics were compared: the yield and transport properties of the contacts. For the contacts evaporated on the top surface, the yield was approximately 60%, whereas for the contacts on the bottom, it reached about 85%. The sheet resistance of Bi_2_Te_3_ was determined from the four-terminal measurements and was about 1–3 ohms. Afterward, we employed two-terminal measurements to assess the resistance of the contacts, calculated as the difference between the resistance obtained from the two-terminal measurement and the resistance of the sample. The average contact resistance was found to be about 1 kohm and 200 ohms for the top and bottom contacts, respectively.

Our result means that additional efforts are required to optimize the all-inert-atmosphere lithographical routes.

### 4.4. (1144) Iron-Based Superconductors and Inert-Atmosphere Transfer to Cryostat

Another application of the glove box is the transfer of air-unstable materials for measurements. Iron-based superconductors containing alkali metals, such as AEuFe_4_As_4_ (where A = Rb, Cs) compounds, known as (1144) superconductors, are prone to rapid degradation due to interaction with oxygen and water [37,38,39].

**Figure 12:**
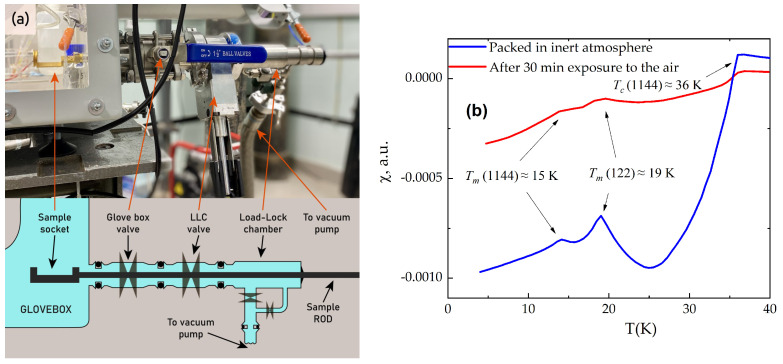
(**a**) Photo (top) and schematic (bottom) of the all-inert-atmosphere transfer module to cryomagnetic measurements; (**b**) magnetic susceptibility of the fresh crystal of EuCsFe_4_As_4_ transferred from the glovebox (blue) and the same crystal exposed to air for 30 min (red).

Through the KF40 port, the samples can be transferred directly for measurements (transport or magnetic susceptibility) in the inert atmosphere. For this purpose, a gate valve is adjusted to the evacuated load lock chamber of the cryostat sample holder, as shown in Figure 12a. We use the Cryogenic load locked system for resistivity measurements and magnetometry. The sample is mounted in the glove box, then moved to the load lock. The gate and glove box valves are closed and the load lock is evacuated. The stick with the load lock and its gate valve is transferred to the gate-valve-terminated cryostat. Figure 12b shows the temperature dependencies of the magnetic susceptibility of a EuCsFe_4_As_4_ single crystal with inclusions of the EuFe_2_As_2_ phase. The crystals of EuCsFe_4_As_4_ were grown by the “self-flux” method and selected from the batch presented in Refs. [37,38]. The crystals were stored in a glove box and then mounted to the three-coil cell for magnetic susceptibility measurements. The measurements of the fresh sample are shown by a blue curve and demonstrate an abrupt superconducting transition at 36 K, as well as the magnetic transition of the Eu subsystem at 15 K, inherent to the (1144) system, and also a magnetic transition at 19 K for the inclusions of the EuFe_2_As_2_ phase. Then, the sample was taken out of the setup and subjected to ambient conditions for 30 min and measured again. It can be seen that the sample exposed to air had degraded (red curve in Figure 12b); all superconductive and magnetic features are smeared.

## 5. Discussion: Further Development

We should note here that building such a complex setup is time consuming. This is especially true for all elements related to leak detection and the joints of different functional blocks. Even if ordered from a commercial company, such a setup will not be functional within a year. This should be taken into account when evaluating resources.

The setup essentially serves as an upgradable technological platform. The most crucial component, which should be especially well designed, is the transfer machine lithographer in the main glove box (the cleanest part of the system). Notably, the transfer machine is not meant for regular disassembly. On the contrary, other elements can be replaced without compromising the clean, oxygen-free environment.

Another important issue is the purity of the gas. A combination of three major factors is known to be fatal for unstable materials: oxygen, humidity, and UV radiation. To reduce UV illumination, we cover the lamp with a yellow filter. We believe that reducing the oxygen content is the most critical aspect. For this purpose, we purchased a glove box with a gas purification system. Maintaining humidity at an optimal level is crucial for a successful polypropylene carbonate-based transfer process [40]. Our practical understanding suggests that 30% humidity is optimal. For some materials (see above examples), the humidity should be zero and can be manually adjusted, considering that water content decreases when introducing new portions of dry gas into the glove box.

To avoid water during the fabrication, a waterless lithography process is required. Such a process is highly in demand but is not typical for UV-visible light photoresists. As an option, one may use a PMMA resist capable of electron lithography. This resist could be spin-coated in the glovebox, and then, the structure could be taken out for exposure and returned back to the inert atmosphere for further processing.

One more issue concerns the quality of the crystals used. It is crucial, especially for thin and monolayer samples in laboratory prototypes, to use the highest-quality crystals. For instance, the transport characteristics in black phosphorus (BP) are vendor-dependent [41]. The feasibility of monolayer exfoliation is also contingent on the choice of the vendor. Therefore, it is imperative to utilize crystals from reputable and trusted suppliers (for example HQGraphene) (Accessed on 30 December 2023).

Note that the motorization of all stages is not just for convenience. In combination with machine vision and machine learning elements, it allows for a controlled search for flakes with the desired properties, similar to Ref. [10]. Potentially, through deep automation and AI technologies, it becomes possible to assemble rather complex devices [42]. Therefore, we believe that motorization is a crucial option.

The presented technology is just for laboratory prototyping needs. However, any useful device from 2D materials should be based on planar technology rather than on the flakes exfoliated from small, individual single crystals. There exists a lot of research activity in the field of forthcoming 2D micro- and nano-electronics [43], including flexible electronics [44,45], sensors [46,47], overcoming the limitations for Moore’s law [48], etc. For graphene and hexagonal boron nitride, cheap CVD growth of large-scale materials is well developed. However, the issue of finite oxygen permeability for wafer-scale hBN membranes, which is vital for unstable material conservation, is not fully resolved yet [49]. For transition metal dichalcogenides, cheap methods of wafer-scale coatings are developed on the basis of MOCVD/MOVPE [50].

**Figure 13:**
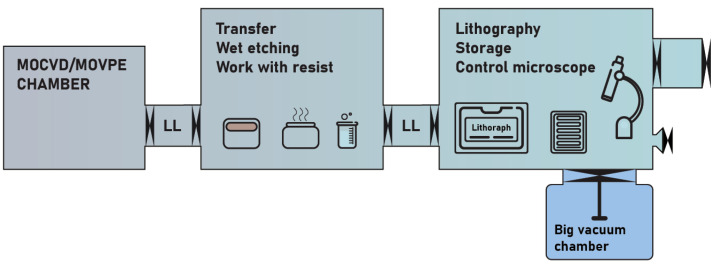
Principal scheme of wafer-scale all-inert-atmosphere 2D materials processing cluster.

We speculate that the presented technology could be upgraded for working with wafer-scale substrates. Schematically, such an extension is shown in Figure 13.

Building up such a cluster starts with the choice of materials and goals. Air-stable 2D layers of graphene [51] and boron nitride [52] could be fabricated outside. Industry-compatible processes for the other 2D materials (MOVPE/MOCVD) require toxic gases and should be adjusted in advance. A chamber with planar layer fabrication should have a port to the inert-atmosphere glove box with a load lock to the liquid processing chamber. Liquid processes include photoresist coating and baking, wet etching, photoresist development, delamination of 2D layers from the substrates, liquid wafer-scale transfer [53], and acid treatment [54] to improve the quality of TMDCs. The atmosphere in this box is basically contaminated by the vapors of various liquids. Similarly to our setup, one should avoid the storage of 2D materials here.

In the third glove box, there should be a lithographer. This is the glove box with the cleanest inert atmosphere. All substrates and 2D materials have, therefore, to be stored here (or in a vacuum). Time-consuming in situ inspection of the wafers (shown by a microscope) should also be placed here. Wafer inspection could be performed either using scanning probe tools (atomic force microscopy, conductance microscopy, Kelvin probe, conductance 4-probe, etc.) or by optical tools (spectroscopy of luminescence or Raman scattering, polarization microscopy). A vacuum system with the proper processes is connected to the glove box through the load lock.

This vacuum system is required for plasma etching, metal evaporation, sputtering, and plasma cleaning of the substrates. It can be customized by numerous suppliers, e.g., Plassys, AJA International, HindHighVac, Kurt J. Leskes, etc. Importantly, it has to provide uniformity over the wafer scale and, therefore, should be rather large. This part may require more than one chamber and its own transfer system. The extreme two-dimensionality of the processed system may, however, soften the uniformity requirements.

The lithographer is the most crucial element. We believe that for cheap and high-throughput operation, this should be a contact mask aligner, because it is a compact lithography device that can potentially be accommodated in the glove box. There are many commercially available table-top solutions, e.g., by Karl Suss, Planar, EVG, MIDAS Systems (All references are accessed on 30 December 2023), etc. Note that the lithographer has to be vibrationally isolated to pattern at the wafer scale with micrometer resolution.

Scanning probe table-top lithographers (e.g., the one by Heidelberg Instruments) are too slow for wafer scale—0.5 mm^2^/min—although they provide sub-micrometer resolution.

Table-top maskless optical lithographers are supplied by many companies, e.g., Raith, MIdalix, Heidelberg Instruments, Kloe (All references are accessed on 30 December 2023), etc. These machines can be a compromise in terms of resolution and throughput ratio. They can pattern arbitrary designs with a speed of up to tens of mm^2^ per minute (with a ∼1 μ m resolution).

## 6. Conclusions

We demonstrated a combined approach to all-inert-atmosphere technology for air-degradable 2D materials. Namely, we add thermal evaporation/plasma processing, wet processes, and lithography to the ubiquitous transfer machine in the glove box. Such modifications are relatively inexpensive because all technological operations are performed with small (typically 1 cm × 1 cm) substrates. The suggested scheme could further be implemented in 2D materials laboratories and opens new possibilities for device prototyping out of unstable two-dimensional materials.

## Figures and Tables

**Table 1:** Approximate costs of all basic elements and possible alternatives. All references are accessed on 30 December 2023.

Block	Component	Our Solution	Our Costs, USD	Recommended Solution	Recommended Cost, USD
Glovebox	Glovebox	https://vilitek.com/	14,000	Aliexpress	6000
	Microscope	Data	Data	Aliexpress	100
	Table	Local supplier	200	The same	200
	Scroll pump	Edwards NXDS 10	5000	Baosi	3500
	Gas tanks, reducers, etc.	Local suppliers	500	The same	500
Small glovebox	Glovebox	Aliexpress	1000	Example	2000
	Table	local supplier	100	The same	100
	Hot Plate	Aliexpress	65	Aliexpress	20
	Spin-coater	Aliexpress	1600	Instras	700
]Vacuum part	ISO100 Chamber	Aliexpress	1000	The same	1000
	Turbopump	Used from eBay	2000	Pfeiffer	7500
	Vacuum furniture	Flanges, feedthroughs, o-rings, clamps, hoses, valves	1000	The same	1000
	Vacuum gauge	Thyracont	1500	The same	1500
	Thermal evaporation	Aliexpress	700	The same	700
	Plasma generator 500 W	Used from eBay	1500	Aliexpress	6500
Transfer lithography	Microscope	Home-made from Aliexpress components	400	The same	400
	XYZ Θ stage	Motorized, used	1000	Manual, Aliexpress	150
	Mask holder	Aliexpress	100	The same	100
	Automation (Raspberry Pi, controllers, AC/DC adapters)	Aliexpress	500	Manual system	100
	Computer	PC with monitor	1100	The same	1100
Total			∼33,000		∼ 32,000

## Data Availability

Data are contained within the article.

## References

[1] Geim A.K., Grigorieva I.V. (2013). Van derWaals Heterostructures. Nature.

[2] Novoselov K., Mishchenko A., Carvalho A., Castro Neto A.H. (2016). 2D materials and van der Waals heterostructures. Science.

[3] Ryu B., Wang L., Pu H., Chan M.K.Y., Chen J. (2022). Understanding, discovery, and synthesis of 2D materials enabled by machine learning. Chem. Soc. Rev..

[4] Wang P., Jia C., Huang Y., Duan X. (2001). Van der Waals Heterostructures by Design: From 1D and 2D to 3D. Matter.

[5] Castellanos-Gomez A., Duan X., Fei Z., Gutierrez H.R., Huang Y., Huang X., Quereda J., Qian Q., Sutter E., Sutter P. (2022). Van der Waals heterostructures. Nat. Rev. Methods Prim..

[6] Chaves A., Azadani J., Alsalman H., Costa D., Frisenda R., Chaves A., Song S., Kim Y., He D., Zhou J. (2020). Bandgap engineering of two-dimensional semiconductor materials. Npj 2D Mater. Appl..

[7] Wang X., Sun Y., Liu K. (2019). Chemical and structural stability of 2D layered materials. 2D Mater..

[8] Cao Y., Mishchenko A., Yu G.L., Khestanova E., Rooney A.P., Prestat E., Kretinin A.V., Blake P., Shalom M.B., Woods C. (2015). Quality Heterostructures from Two-Dimensional Crystals Unstable in Air by Their Assembly in Inert Atmosphere. Nano Lett..

[9] Gant P., Carrascoso F., Zhao Q., Ryu Y.K., Seitz M., Prins F., Frisenda R., Castellanos-Gomez A. (2020). A system for the deterministic transfer of 2D materials under inert environmental conditions. 2D Mater..

[10] Masubuchi S., Morimoto M., Morikawa S., Onodera M., Asakawa Y., Watanabe K., Taniguchi T., Machida T. (2018). Autonomous robotic searching and assembly of two-dimensional crystals to build van der Waals superlattices. Nat. Commun..

[11] Buapan K., Somphonsane R., Chiawchan T., Ramamoorthy H. (2021). Versatile, Low-Cost, and Portable 2D Material Transfer Setup with a Facile and Highly Efficient DIY Inert-Atmosphere Glove Compartment Option. ACS Omega.

[12] Guo S., Luo M., Shi G., Tian N., Huang Z., Yang F., Ma L., Wang N.Z., Shi Q., Xu K. (2023). An ultra-high vacuum system for fabricating clean two-dimensional material devices. Rev. Sci. Instrum..

[13] Masubuchi S., Sakano M., Tanaka Y., Wakafuji Y., Yamamoto T., Okazaki S., Watanabe K., Taniguchi T., Li J., Ejima H. (2022). Dry pick-and-flip assembly of van der Waals heterostructures for microfocus angle-resolved photoemission spectroscopy. Sci. Rep..

[14] Frisenda R., Navarro-Moratalla E., Gant P., De Lara D.P., Jarillo-Herrero P., Gorbachev R.V., Castellanos-Gomez A. (2018). Recent progress in the assembly of nanodevices and van der Waals heterostructures by deterministic placement of 2D materials. Chem. Soc. Rev..

[15] Guo H.-W., Hu Z., Liu Z.-B., Tian J.-G. (2021). Stacking of 2D Materials. Adv. Funct. Mater..

[16] Gray M.J., Kumar N., O’Connor R., Hoek M., Sheridan E., Doyle M.C., Romanelli M.L., Osterhoudt G.B., Wang Y., Plisson V. (2020). A cleanroom in a glovebox. Rev. Sci. Instrum..

[17] Wei Z., Liao M., Guo Y., Tang J., Cai Y., Chen H., Wang Q., Jia Q., Lu Y., Zhao Y. (2020). Scratching lithography for wafer-scale MoS_2_ monolayers. 2D Mater..

[18] Rao Q., Gao G., Wang X., Xue H., Ki D.-K. (2023). Scratching lithography, manipulation, and soldering of 2D materials using microneedle probes. arXiv.

[19] Jung Y., Choi M.S., Nipane A., Borah A., Kim B., Zangiabadi A., Taniguchi T., Watanabe K., Yoo W.J., Hone J. (2019). Transferred via contacts as a platform for ideal two-dimensional transistors. Nat. Electron..

[20] Satterthwaite P., Zhu W., Jastrzebska-Perfect P., Tang M., Spector S., Gao H., Kitadai H., Lu A., Tan Q., Tang S. (2023). Van der Waals device integration beyond the limits of van der Waals forces using adhesive matrix transfer. Nat. Electron..

[21] Pugachev M., Duleba A., Galiullin A., Kuntsevich A. (2021). Micromask Lithography for Cheap and Fast 2D Materials Microstructures Fabrication. Micromachines.

[22] Gogotsi Y., Anasori B. (2019). The Rise of MXenes. ACS Nano.

[23] Botana A.S., Norman M.R. (2019). Electronic structure and magnetism of transition metal dihalides: Bulk to monolayer. Phys. Rev. Mater..

[24] Mogulkoc A., Modarresi M., Rudenko A.N. (2020). Two-dimensional chromium pnictides CrX (X = P, As, Sb): Half-metallic ferromagnets with high Curie temperature. Phys. Rev. B.

[25] Chen Y., Sun Y., Peng J., Tang J., Zheng K., Liang Z. (2018). 2D Ruddlesden–Popper Perovskites for Optoelectronics. Adv. Mater..

[26] Jain A., Bharadwaj P., Heeg S., Parzefall M., Taniguchi T., Watanabe K., Novotny L. (2018). Minimizing residues and strain in 2D materials transferred from PDMS. Nanotechnology.

[27] Zhao Q., Wang T., Ryu Y.K., Frisenda R., Castellanos-Gomez A. (2020). An inexpensive system for the deterministic transfer of 2D materials. J. Phys. Mater..

[28] Martanov S.G., Zhurbina N.K., Pugachev M.V., Duleba A.I., Akmaev M.A., Belykh V.V., Kuntsevich A.Y. (2020). Making van der Waals Heterostructures Assembly Accessible to Everyone. Nanomaterials.

[29] Luo W., Zemlyanov D., Milligan C., Du Y., Yang L., Wu Y., Ye P. (2016). Surface chemistry of black phosphorus under a controlled oxidative environment. Nanotechnology.

[30] Favron A., Gaufrès E., Fossard F., Phaneuf-L’Heureux A., Tang N., Lévesque P., Loiseau A., Leonelli R., Francoeur S., Martel R. (2015). Photooxidation and quantum confinement effects in exfoliated black phosphorus. Nat. Mater..

[31] Kuntz K., Wells R., Hu J., Yang T., Dong B., Guo H., Woomer A., Druffel D., Alabanza A., Tománek D. (2017). Control of Surface and Edge Oxidation on Phosphorene. Acs Appl. Mater. Interfaces.

[32] Ribeiro H.B., Pimenta M.A., de Matos C.J.S. (2018). Raman spectroscopy in black phosphorus. J. Raman Spectrosc..

[33] Perello D., Chae S., Song S., Lee Y. (2015). High-performance n-type black phosphorus transistors with type control via thickness and contact-metal engineering. Nat. Commun..

[34] Radisavljevic B., Kis A. (2013). Mobility engineering and a metal–insulator transition in monolayer MoS_2_. Nat. Mater..

[35] Kumar A., Viscardi L., Faella E., Giubileo F., Intonti K., Pelella A., Sleziona S., Kharsah O., Schleberger M., Di Bartolomeo A. (2023). Temperature dependent black phosphorus transistor and memory. Nano Express.

[36] Li L., Yu Y., Ye G.J., Ge Q., Ou X., Wu H., Feng D., Chen X.H., Zhang Y. (2014). Black phosphorus field-effect transistors. Nat. Nanotechnol..

[37] Kuzmichev S.A., Pervakov K.S., Vlasenko V.A., Degtyarenko A.Y., Gavrilkin S.Y., Kuzmicheva T.E. (2022). Andreev Spectroscopy of EuCsFe4As4 Stoichiometric Superconducting Pnictide. JETP Lett..

[38] Degtyarenko A.Y., Vlasenko V.A., Kuzmicheva T.E., Pervakov K.S., Gavrilkin S.Y., Tsvetkov A.Y., Kuzmichev S.A. (2023). Anisotropy of the Critical Current and Abrikosov Vortex Pinning in Magnetic Superconductor EuCsFe_4_As_4_. JETP Lett..

[39] Degtyarenko A.Y., Karateev I.A., Ovcharov A.V., Vlasenko V.A., Pervakov K.S. (2022). Synthesis and HRTEM Investigation of EuRbFe_4_As_4_ Superconductor. Nanomaterials.

[40] Pizzocchero F., Gammelgaard L., Jessen B.S., Caridad J.M., Wang L., Hone J., Bøggild P., Booth T.J. (2016). The hot pick-up technique for batch assembly of van der Waals heterostructures. Nat. Commun..

[41] Welch K., Doha M., Uttley Z., Fereidouni A., Omolewu A., Santos J., El-Shenawee M., Churchill H. Comparison of Hall Mobility and Carrier Density of Thin Black Phosphorus Exfoliated from Bulk Crystals Provided by Various Vendors. Proceedings of the 2022 IEEE USNC-URSI Radio Science Meeting (Joint With AP-S Symposium).

[42] Mannix A., Ye A., Sung S., Ray A., Mujid F., Park C., Lee M., Kang J., Shreiner R., High A. (2022). Robotic four-dimensional pixel assembly of van der Waals solids. Nat. Nanotechnol..

[43] Jia L., Wu J., Zhang Y., Qu Y., Jia B., Chen Z., Moss D.J. (2022). Fabrication Technologies for the On-Chip Integration of 2D Materials. Small Methods.

[44] Joe D.J., Park E., Kim D.H., Doh I., Song H.-C., Kwak J.Y. (2023). Graphene and Two-Dimensional Materials-Based Flexible Electronics for Wearable Biomedical Sensors. Electronics.

[45] Yang D., Wang H., Luo S., Wang C., Zhang S., Guo S. (2019). Paper-Cut Flexible Multifunctional Electronics Using MoS_2_ Nanosheet. Nanomaterials.

[46] Hywel M., Rout C.S., Late D.J. (2019). Fundamentals and Sensing Applications of 2D Materials.

[47] Rehman A., Park S.-J. (2020). State of the art two-dimensional materials-based photodetectors: Prospects, challenges and future outlook. J. Ind. Eng. Chem..

[48] Schram T., Sutar S., Radu I., Asselberghs I. (2022). Challenges of Wafer-Scale Integration of 2D Semiconductors for High-Performance Transistor Circuits. Adv. Mater..

[49] Ko H., Choi S., Kim J., Kim Y., Kim Y., Adofo L., Jung M., Kim Y., Jeong M., Kim K. (2021). Toward non-gas-permeable hBN film growth on smooth Fe surface. 2D Mater..

[50] Xu X., Guo T., Kim H., Hota M.K., Alsaadi R.S., Lanza M., Zhang X.H.N. (2022). Alshareef Growth of 2D Materials at the Wafer Scale. Adv. Mater..

[51] Baojun Sun B., Pang J., Cheng Q., Zhang S., Li Y., Zhang C., Sun D., Ibarlucea B., Li Y., Chen D. (2021). Synthesis of Wafer-Scale Graphene with Chemical Vapor Deposition for Electronic Device Applications. Adv. Mater. Technol..

[52] Park J.-H., Park J.C., Yun S.J., Kim H., Luong D.H., Kim S.M., Choi S.H., Yang W., Kong J., Kim K.K. (2014). Large-area monolayer hexagonal boron nitride on Pt foil. ACS Nano.

[53] Watson A.J., Lu W., Guimarães M.H.D., Stöhr M. (2021). Transfer of large-scale two-dimensional semiconductors: Challenges and developments. 2D Mater..

[54] Han H., Lu A., Lu L., Huang J., Hsu C., Lin Y., Chiu M., Suenaga K., Chu C., Kuo K. (2016). Photoluminescence Enhancement and Structure Repairing of Monolayer MoSe_2_ by Hydrohalic Acid Treatment. ACS Nano.

